# Glutamine May Repress the Weak LPS and Enhance the Strong Heat Shock Induction of Monocyte and Lymphocyte HSP72 Proteins but May Not Modulate the HSP72 mRNA in Patients with Sepsis or Trauma

**DOI:** 10.1155/2015/806042

**Published:** 2015-10-13

**Authors:** Efrossini Briassouli, Marianna Tzanoudaki, Dimitris Goukos, Christina Routsi, Serafim Nanas, Kostas Vardas, Kleovoulos Apostolou, Maria Kanariou, George Daikos, George Briassoulis

**Affiliations:** ^1^First Department of Propaedeutic Internal Medicine, University of Athens, Athens, Greece; ^2^Department of Immunology-Histocompatibility, Specialized Center & Referral Center for Primary Immunodeficiencies-Paediatric Immunology, “Aghia Sophia” Children's Hospital, Athens, Greece; ^3^First Critical Care Department, Evangelismos Hospital, University of Athens, Athens, Greece; ^4^Pediatric Intensive Care Unit, University Hospital, University of Crete, 71500 Heraklion, Greece

## Abstract

*Objective*. We assessed the lipopolysaccharide (LPS) or heat shock (HS) induction of heat shock protein-72 (HSP72) in peripheral blood mononuclear cells (PBMCs) of patients with severe sepsis (SS) or trauma-related systemic inflammatory response syndrome (SIRS), compared to healthy individuals (H); we also investigated any pre- or posttreatment modulating glutamine (Gln) effect. *Methods*. SS (11), SIRS (10), and H (19) PBMCs were incubated with 1 *μ*g/mL LPS or 43°HS. Gln 10 mM was either added 1 h before or 1 h after induction or was not added at all. We measured monocyte (m), lymphocyte (l), mRNA HSP72, HSP72 polymorphisms, interleukins (ILs), monocyte chemoattractant protein-1 (MCP-1), and cortisol levels. *Results*. Baseline lHSP72 was higher in SS (*p* < 0.03), and mHSP72 in SIRS (*p* < 0.02), compared to H. Only HS induced l/mHSP72/mRNA HSP72; LPS induced IL-6, IL-8, IL-10, and MCP-1. Induced mRNA was related to l/mHSP72, and was related negatively to cytokines. Intracellular l/mHSP72/HSP72 mRNA was related to serum ILs, not being influenced by cortisol, illness severity, and HSP72 polymorphisms. Gln did not induce mRNA in any group but modified l/mHSP72 after LPS/HS induction unpredictably. *Conclusions*. HSP72 mRNA and l/mHSP72 are higher among critically ill patients, further induced by HS, not by LPS. HSP72 proteins and HSP72 mRNA are related to serum ILs and are negatively related to supernatant cytokines, not being influenced by HSP72 polymorphisms, cortisol, or illness severity. Gln may depress l/mHSP72 after LPS exposure and enhance them after HS induction, but it may not affect early induced HSP72 mRNA.

## 1. Introduction

Fever commonly characterizes the infectious or noninfectious systemic inflammatory response syndrome (SIRS) [1]. For patients with sepsis, there may be a relationship between elevated early peak temperature and decreased mortality at 28 days [2] or hospital discharge [3]. In trauma patients with SIRS, however, high fever (>38°C) and fever burden in the first 72 h are associated with an increase in mortality [4]. Furthermore, fever treatment of influenza infection has been reported to be associated with mortality [5]. In intensive care unit (ICU) patients without acute neurological injury, however, no association between antipyretics and mortality could be found [6].

Fever is activating immune cells by inducing antibody synthesis and cytokine release [7]. Additionally, the cell responds to various external and internal stressors by generating complex intracellular heat shock proteins (HSPs)/interleukin (IL) networks [8]. HSP72, a 72 kDa highly inducible HSP [4], is believed to effectively protect against a potentially lethal heat shock (HS) [9]. In vivo or in vitro induction of HSP72 [10] was shown to be associated with cytoprotective effects against inflammation, lipopolysaccharide (LPS), and fever [11] through molecular chaperone-like modulatory effect [12].

SIRS may induce an HS factor-1 (HSF-1) competitive inhibition of nuclear factor-*κ*B (NF-*κ*B) nuclear binding and prevent NF-*κ*B from being released from its complex with I*κ*B*α*, seriously affecting innate immunity [13]. Glutamine (Gln) may be a potent enhancer of the regulatory HSF-1 expression and HSF-1 transcription activity, directly manipulating NF-*κ*B activation [14], inducing the HSP72 expression [15] and or attenuating cytokine release in cultured peripheral blood mononuclear cells (PBMCs) [16]. Thus, parenteral Gln in ICU patients significantly increased serum HSP72, possibly associated with improved outcome [17]. Recent multicentre studies in adults and children, however, could not verify that early provision of L-alanyl-glutamine dipeptide may exert any specific beneficial effect [18]. In contrast, Gln has been associated with worse outcome when given to critically ill patients with renal and/or other organ failures [19]. Similarly, clinical randomized studies in mechanically ventilated ICU patients nutritionally supported with Gln enriched formulae either did not improve clinical outcome [20] or even increased adjusted mortality at six months [21].

We have recently shown that LPS does not promote or inhibit extracellular HSP72 secretion in supernatants of PBMCs, the most sensitive blood cells for HSP72 expression in critically ill patients [22]. We have also shown that Gln does not induce any of the Th1, Th2, and Th17 cytokines, although it may initially suppress HSP72 in either healthy or septic human PBMCs [23]. It has been previously shown, however, that mHSP72 response is dependent on intracellular HSP72 amount and severity of in vitro stress in healthy male volunteers [22]. In vivo, serum cortisol, IL-6, IL-10, and extracellular HSP72 levels were higher and intracellular PBMCs' HSP72 lower in severe sepsis (SS) compared to H [24]. In addition, genetic variants of the HSP72 genes may also affect HSP72 induction, indirectly influencing inflammation, infection severity, and development of septic shock [25].

We now sought to evaluate HSP72 mRNA fold changes and intracellular HSP72 levels in monocytes (mHSP72) or lymphocytes (lHSP72) during LPS exposure or HS recovery of PBMCs. Using our previously established PBMC model of LPS and Gln net effects [23], we first compared any inductive and or repressive effect of HS with that of LPS on PBMCs' HSP72 mRNA, mHSP72, and lHSP72 in patients with SS or trauma-related SIRS and healthy individuals (H). Secondly, we investigated pre- or posttreatment Gln modulation of any possible HS or LPS induction or repression effect on PBMCs' HSP72. We hypothesized that baseline mHSP72 or lHSP72 protein expression could have already been induced in ICU patients, differently affected by LPS and HS and possibly differently modified by Gln. Furthermore, we hypothesized that HSP72 simple nucleotide polymorphisms (SNPs), severity of illness, and inflammatory hormonal response might also interfere with the HS or LPS induction of mRNA or intracellular HSP72 proteins in stimulated PBMCs.

## 2. Materials and Methods

### 2.1. Subjects

Eleven patients with severe sepsis/septic shock (SS group), 10 patients with trauma-related SIRS, and 19 healthy volunteers participated in this study. The study was approved by the Medical School, University of Athens, and by the Hospital Ethics Committee of Evangelismos Hospital. Written informed consent was provided by patients' relatives and volunteers. Septic shock and severe sepsis were defined according to the criteria of the American College of Chest Physicians/Society of Critical Care Medicine consensus conference [26]. Patients were included in the first 48 hours after the first organ failure (severe sepsis) or hypotension not responding to fluids (septic shock).

### 2.2. Sample Collection

Twenty mL of blood was drawn between 8:00 and 9:00 o'clock in the morning in a sterile anticoagulated tube containing heparin (approximately 15 USP units of heparin per milliliter of blood). A healthy control sample was simultaneously drawn and processed with every patient sample. Blood was processed within one hour after collection.

### 2.3. Cell Culture Protocol

To compare possible induction and/or repression effects of LPS with those of HS on PBMCs' HSP72 expression in patients with SS or SIRS and healthy controls, we first isolated fresh PBMCs by gradient centrifugation (Biocoll, Biochrom, Germany). We counted the concentration and the viability of the cells by flow cytometry, using 7AAD, CD45, and CD3 labeling in combination with absolute counting fluorospheres (Beckman Coulter, Miami, FL, USA). This viability test was performed in every measurement before the cell culture and randomly afterwards in order to establish that the cells survived the treatment and no adverse effects were detected. We incubated the cells at a concentration of 10^6^ cells/mL in Roswell Park Memorial Institute Medium (*RPMI 1640 w/o Glutamine*,* Gibco*), containing 5% Fetal Bovine Serum (Biosera, South America) and 1% penicillin-streptomycin at 37°C in a humidified incubator containing 5% CO_2_, using 96-well culture plates. Cells were stimulated either with LPS (final concentration 1 *μ*g/mL) or with heat shock (30 min in 43° water bath) or were left untreated. We used 43 °C heat-shocked cells previously shown to reduce expression of human tissue factor specific mRNA surface protein and activity induced by LPS-stimulated endothelial cells [27].

We added L-Ala-Gln (*Dipeptiven*;* Fresenius-Kabi*, Bad Homburg, Germany) at a final concentration of 10 mM either 1 h before stimulation or 1 h after stimulation or we did not add it at all. The 10 mM dose was chosen because it clinically approximates concentrations of Gln achieved locally during enteral infusion of Gln [28] or plasma concentrations after Gln infusion 0.75 g/kg [29]. At 10 mM L-Ala-Gln induces maximal HSP expression associated with cytokine attenuation in vitro and in vivo [16]. Cells were incubated at 37°C for 4 hours after the HS or the LPS stimulation with or without Gln before or after treatment and were subsequently harvested for intracellular HSP72 staining and flow cytometric analysis. The incubation period was set at four hours because we have previously shown that Gln at 10 mM represses HSP72 in PBMCs of septic or controls by 4 hours without inducing any of the Th1, Th2, and Th17 cytokines (pilot time-dose response and safety study) [23]. We have also shown that LPS at 1 *μ*g/mL induces IL-6 and IL-10 from PBMCs promoting different IL-6 responses in healthy individuals and PBMCs of clinically severely septic patients (time-dose response pilot study) [23].

### 2.4. Intracellular HSP72 Staining and Flow Cytometric Analysis

After staining for surface antigens CD33 PE/Cy5 (clone WM33, BioLegend, San Diego, CA, USA) and CD45 PE/Cy7 (clone HI30, BioLegend, San Diego, USA), cells were fixed and permeabilized and finally stained for intracellular HSP72-FITC (clone C92F3A-5 Enzo Life Sciences, Ann Arbor, MI, USA). HSP90*α*-PE intracellular staining was simultaneously performed (data not shown). All assays were performed according to the manufacturers' instructions. Flow cytometric analysis was performed on an FC-500 instrument (Beckman Coulter, Miami, FL, USA), using CXP software (Beckman Coulter, Miami, FL, USA). PBMCs were identified by CD45 expression versus sideward scatter properties. Monocyte and lymphocyte populations were further identified based on CD33 and CD45 expression intensity. Intracellular HSP72 expression intensity was assessed in each of the aforementioned populations separately, using Mean Fluorescence Intensity (MFI). Quality control of the instrument was regularly performed to ensure Photomultiplier (PMT) voltage stability.

### 2.5. Gene Expression Assays

#### 2.5.1. Stimulation of PBMC

1 × 10^6^ PBMCs were seeded in 24-well cell culture plates in 1 mL of Gln-free RPMI-1640 medium supplemented with 10% FBS and 1% penicillin-streptomycin. The stimulation protocols were as follows: (1) no stimulation, (2) 1 *μ*g/mL LPS (*Escherichia coli* O111:B4, Sigma, St Louis, MO, USA), (3) 1 *μ*g/mL LPS 1 hour after the addition of 10 mM L-Ala-Gln dipeptide (Dipeptiven; Fresenius-Kabi, Bad Homburg, Germany), (4) 1 *μ*g/mL LPS 1 hour before the addition of 10 mM L-Ala-Gln dipeptide (Dipeptiven; Fresenius-Kabi, Bad Homburg, Germany), (5) HS at 43°C for 30 min, (6) HS at 43°C for 30 min following the addition of 10 mM L-Ala-Gln dipeptide, and (7) HS at 43°C for 30 min and addition of 10 mM L-Ala-Gln dipeptide 1 hour afterwards. All stimulations took place in a humidified incubator containing 5% CO_2_ at 37°C. Cells were harvested 2 hours after the final stimulation.

#### 2.5.2. RNA Isolation from PBMC

PBMCs were harvested from the wells to 1.5 mL microcentrifuge tubes and centrifuged at 1500 rpm for 5 min. The supernatant was discarded and the cells homogenized with 750 *μ*L TRIzol reagent (Ambion, Carlsbad, CA, USA). RNA was separated with chloroform and precipitated with isopropanol. Finally, the RNA was washed with 75% alcohol and resuspended in prewarmed, nuclease-free H_2_O. The concentration of the RNA was measured with Qubit RNA Assay Kit (Invitrogen, Eugene, OR, USA) according to manufacturer's instructions on the Qubit 2.0 Fluorometer (Invitrogen, Eugene, OR, USA). RNA integrity was confirmed by agarose gel electrophoresis. All total RNA samples were treated with DNase I, Amplification Grade (Invitrogen, Eugene, OR, USA) to diminish possible genomic DNA contamination.

#### 2.5.3. Reverse Transcription and Quantitative PCR

100 ng of total RNA was reverse-transcribed with Transcriptor High Fidelity cDNA Synthesis Kit (Roche Diagnostics GmbH, Mannheim, Germany) in accordance with the manufacturer's instructions. Briefly, 60 *μ*M random hexamer primers were used with total RNA, 1 mM each of dNTPs, 1x reaction buffer, 20 U RNase inhibitor, and 10 U of Transcriptor High Fidelity Reverse Transcriptase in a total volume of 20 *μ*L. The following thermal cycling conditions were used: 25°C for 10 min, 60°C for 60 min, and 85°C for 5 min.

Complementary DNA (cDNA) was amplified by PCR with the use of 1x Maxima SYBR Green qPCR Master Mix (Thermo Scientific, Lithuania, EU) and 0.2 *μ*M of each primer in a total volume of 20 *μ*L. For the heat shock 70 kDa protein 1A (gene symbol HSP72, amplicon size (bp) 183) the sequences of the primers were as follows: 5′-3′F:CCGAGAAGGACGAGTTTGAG; R:AATCTTGGAAAGGCCCCTAA. Reaction mixtures were incubated in a Biorad CFX96, C1000 thermal cycler according to the following thermal conditions: initial denaturation at 95°C for 10 min followed by 40 cycles of 95°C for 15 sec and 63°C for 60 sec. Fluorescence emitted by SYBR Green was measured at the end of each cycle. A melting curve analysis followed amplification of cDNA to determine nonspecific amplification. Relative quantification of the target genes was calculated by the ΔΔCt method using B2M as a reference gene.

### 2.6. Supernatant Cytokines

Cell culture supernatants were collected 4, 8, 24, and 48 hours after stimulation and were kept in a temperature of −80°C (data on 4, 8, and 48 hours not shown). Measurement of cytokine levels in supernatants was performed using a multiplex bead-based immunoassay for flow cytometry (Cytometric Bead Array, Becton Dickinson). Namely, the samples were incubated with the appropriate mix of antibody coated fluorescent beads and then with fluorochrome conjugated monoclonal antibodies to the measured proteins. Samples were acquired in a dual LASER flow cytometer (FACS Array, Becton Dickinson) and analyzed with the FCAP Array v3.0 Software.

### 2.7. Serum Cytokines

Serum levels of IL-6, IL-10, IL-17, and IFN-*γ* were analyzed by ELISA assay according to the manufacturer' instructions (Invitrogen, Carlsbad, CA, USA). The interassay and intra-assay CV for each analyte were as follows: 6.2 and 7.8 for IL-6, 3.25 and 2.75 for IL-10, 3.7 and 3.5 for IL-17, and 7.3 and 7.1 for IFN-*γ*. The sensitivities of the assays were <2 pg/mL for IL-6, <1 pg/mL for IL-10, 2 pg/mL for IL-17, and 0.03 IU/mL for IFN-*γ*.

### 2.8. Cortisol

Blood was drawn between 8:00 and 9:00 a.m. in the first 48 h after ICU admission. Blood was collected in tubes containing clot and gel for serum separation and centrifuged at 4°C and serum was stored at −80°C until measurement. Serum cortisol levels were determined using the ADVIA Centaur Immunoassay Analyzer (Siemens Healthcare Diagnostics, Tarrytown, NY, USA).

### 2.9. Genotyping for HSP72 Polymorphic SNPs

Buffy coat was isolated from EDTA-treated blood samples and used for the extraction of genomic DNA with QIAamp DNA Blood Mini Kit (Qiagen Pty Ltd., Victoria, Australia). Genotyping of the polymorphic HSP72 rs6457452 and rs1061581 SNPs was performed by sequencing the promoter region of HSP72 according to the manufacturer's instructions. HSP72 rs6457452 and rs1061581 alleles were determined by restriction fragment length polymorphism polymerase chain reaction (PCR) as previously described [30].

### 2.10. Statistical Analysis

All results were analyzed using SPSS software (version 22.0, SPSS, Chicago, IL) and are presented as mean ± standard error (SE) of mean and median (interquartile range) as appropriate. Nonparametric group comparisons were performed using the Kruskal-Wallis test; all values, including outliers, were analyzed. Any variables that showed differences among groups were subsequently compared by the Mann-Whitney test. Paired differences for continuous variables in the same subjects were analyzed using Wilcoxon signed-rank test. Between-group comparisons were conducted using *χ* ^2^ test for categorical parameters and Spearman's rank correlation coefficient for correlation between two continuous variables. The level of significance between groups was set at *p* < 0.05.

## 3. Results

### 3.1. Differences between Groups in Baseline mRNA (2 Hours without Any Stimulation) and Protein HSP72 Expression (4 Hours without Any Stimulation)

**Figure 1:**
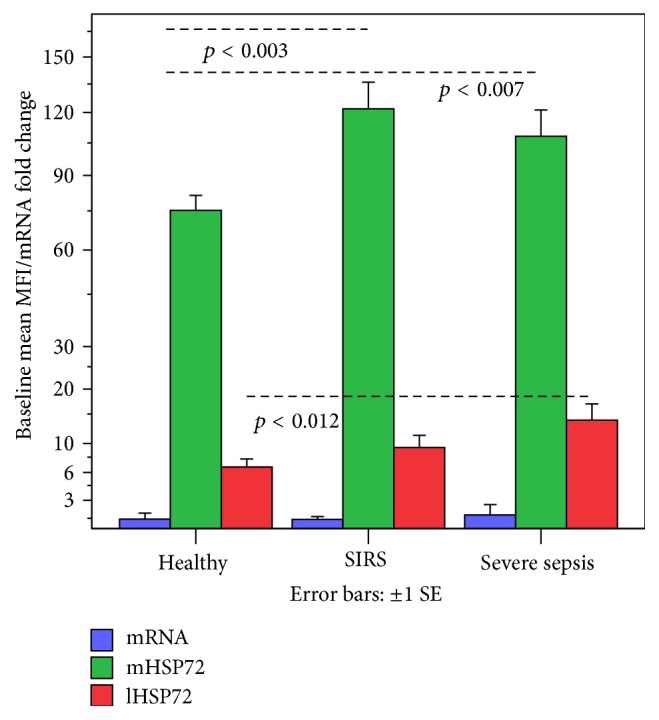
At 4 hours without any stimulation, baseline mHSP72 was higher in SS and SIRS and lHSP72 in SS compared to H. At 2 hours without any stimulation, mRNA showed a nonsignificant increased trend in SS compared to H.

Baseline mHSP72 (*p* < 0.002) and lHSP72 (*p* < 0.008) differed among groups (*Kruskal-Wallis test*). Baseline mHSP72 was higher in SS and SIRS compared to H (*Mann-Whitney U tests*), and lHSP72 was higher in SS compared to H (Figure 1). Although baseline mRNA showed a similar trend for increased expression in SS compared to H and SIRS groups, this difference did reach statistical significance.

### 3.2. Differences of Intracellular Lymphocyte and Monocyte HSP72 Proteins between HS and LPS Induction in H, SIRS, and SS Groups (Mann-Whitney *U* Tests)

**Figure 2:**
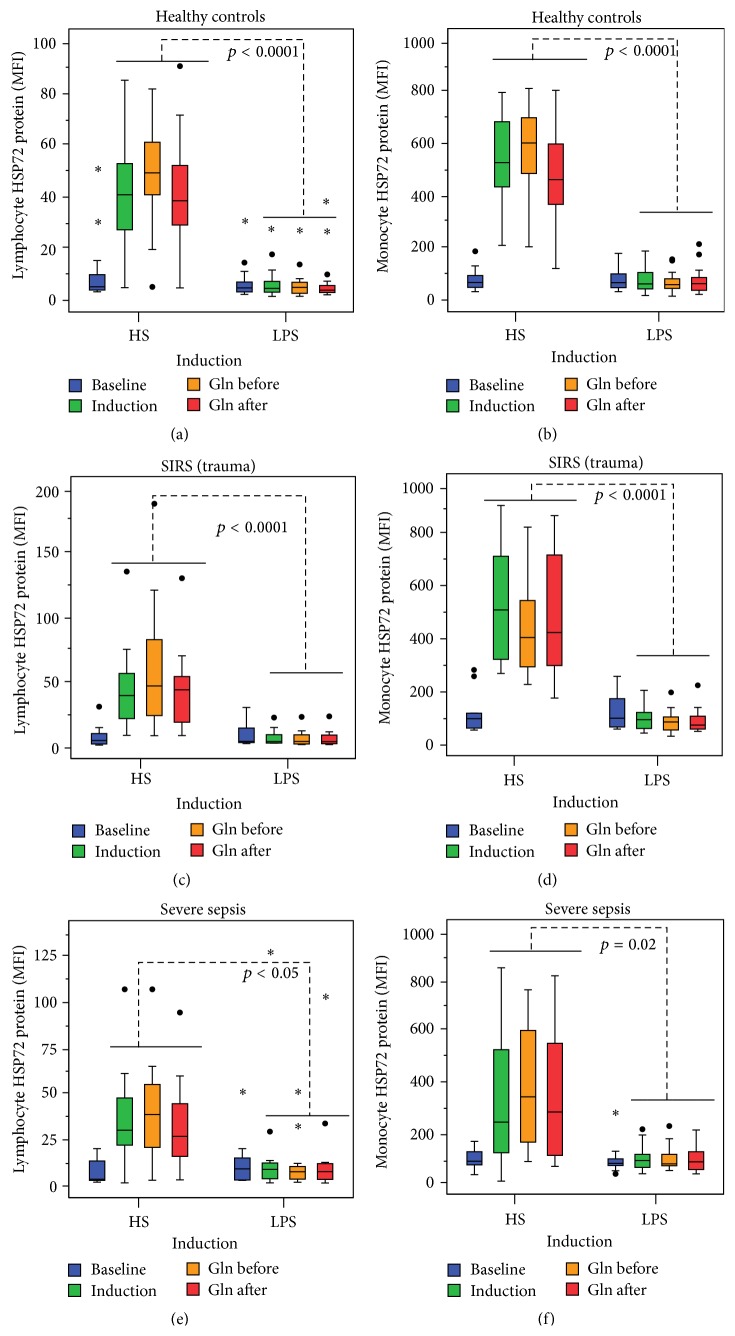
HS alone or with Gln-induced lHSP72 or mHSP72 compared to LPS in healthy individuals (a-b) in patients with SIRS (c-d) or with severe sepsis (e-f). The box and whisker plots show the median (horizontal line within the box) and the 25th and 75th percentiles (whiskers minimum–maximum values). Solid circles represent outliers and stars extremes.

Only HS, either alone or when Gln was given before or after HS, induced lHSP72 or mHSP72 compared to LPS in healthy individuals (Figures 2(a)-2(b)), SIRS (Figures 2(c)-2(d)), or patients with SS (Figures 2(e)-2(f)).

### 3.3. Differences of PBMCs' HSP72 mRNA and Supernatant Cytokines between HS and LPS Induction in H, SIRS, and SS Groups (Mann-Whitney* U* Tests) and Correlations of mRNA with HPS72 Proteins and Cytokines

**Figure 3:**
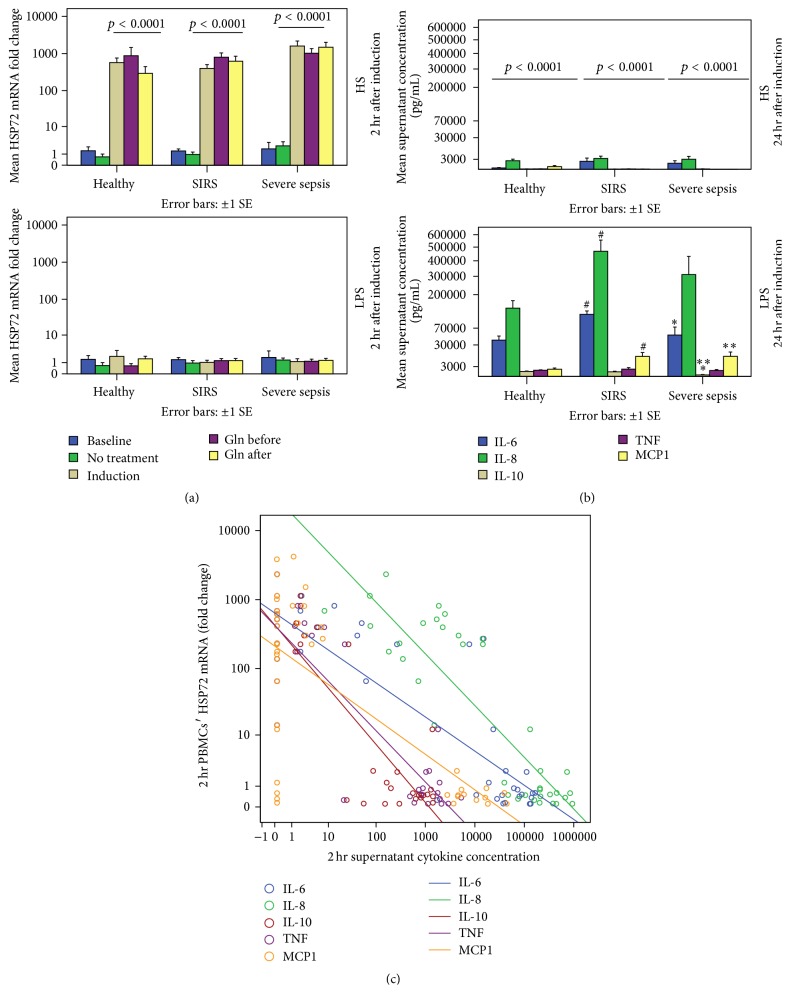
PBMCs' HSP72 mRNA was significantly induced by HS within 2 hours compared to the LPS induction (a). Dashed lines with *p* values indicate differences between the two induction modalities in each group. Baseline and various treatment models did not differ among H, SIRS, and SS groups in either HS or LPS induction modes. On the contrary, 24 hr supernatant cytokines were induced by LPS and not by HS, especially among critically ill patients (b). Dashed lines with *p* values indicate differences between the two induction modes in each group. Cytokines differed among groups in the LPS induction mode. Differences are indicated between groups by #, SIRS versus H, *∗*, SS versus H, and *∗∗*, SIRS versus SS. Induced mRNA was positively related to the intracellular HSP72 proteins (*p* < 0.0001). In contrast it was negatively related (*p* < 0.0001) to IL-6, IL-8, IL-10, TNF, and MCP-1 (c).

Paralleling HS-induced intracellular HSP72, PBMCs' mRNA has already been induced by HS within 2 hours compared to the LPS induction (Figure 3(a)). Contrasting the HSP72 mRNA and protein intracellular PBMCs' expressions, supernatant cytokines were induced by LPS rather than by HS (Figure 3(b)). In response to LPS, 24-hour supernatant IL-6, IL-8, IL-10, and MCP-1 were increased among PBMCs of critically ill patients compared to those of H, with a better IL-6 and IL-10 response in SIRS compared to SS (Figure 3(b)). In accordance with these opposing results, induced mRNA was positively related to intracellular lHSP72 (*r*_*s*_ = 0.65, *p* < 0.0001) and mHSP72 (*r*_*s*_ = 0.70, *p* < 0.0001) but was negatively related to supernatant IL-6 (*r*_*s*_ = −0.77, *p* < 0.0001), IL-8 (*r*_*s*_ = −0.81, *p* < 0.0001), IL-10 (*r*_*s*_ = −0.73, *p* < 0.0001), TNF (*r*_*s*_ = −0.77, *p* < 0.0001), and MCP-1 (*r*_*s*_ = −0.54, *p* < 0.0001) (Figure 3(c)).

### 3.4. Related Samples Wilcoxon Signed-Rank Tests of lHSP72 and mHSP72 Proteins between Gln Treatment Regimens and HS or LPS Induction Methods in H, SIRS, and SS Groups

**Figure 4:**
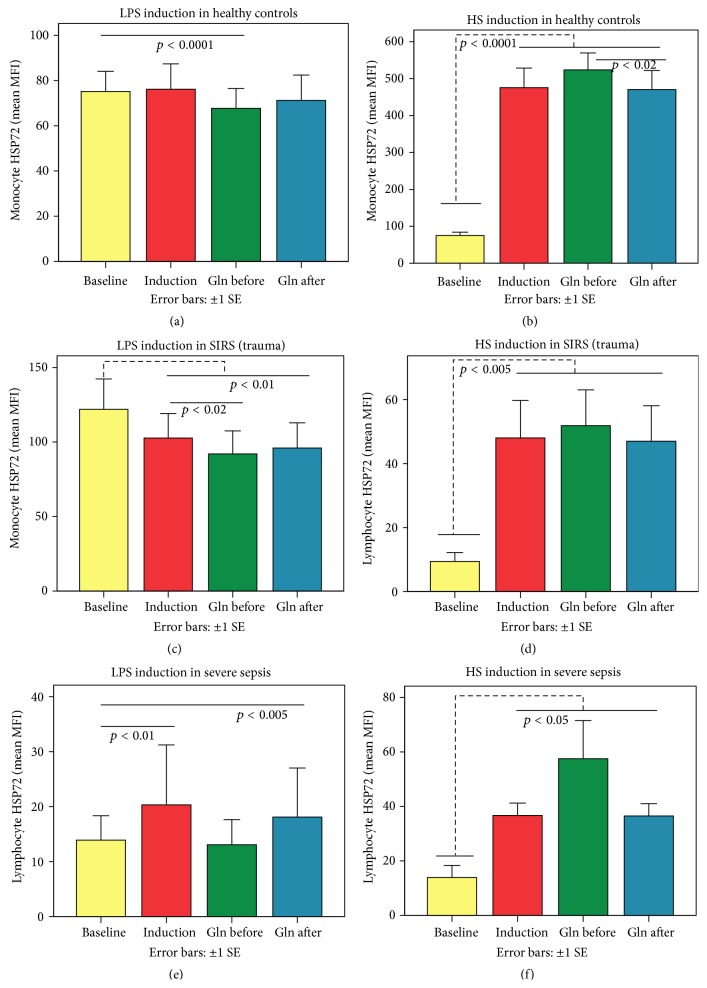
In healthy controls only LPS pretreated with Gln suppressed mHSP72 baseline expression (a). Gln given 1 hour before HS further enhanced the induced mHSP72 expression in comparison to Gln given 1 hour after induction (b). In SIRS Gln given 1 hour before LPS further suppressed the already LPS-suppressed mHSP72 baseline expression (c); in the HS induction model, Gln given before HS showed a trend to increase further the already induced lHSP72 expression (d). In septic PBMCs, only Gln given before LPS abolished the LPS induction effect on lHSP72 (e). However, Gln also given before and not after HS further induced lHSP72 baseline expression (f). These differences between HS and LPS and between Gln given before or after induction clearly underline a Gln pretreatment depressive trend to lymphocyte and monocyte HSP72 proteins of PBMCs exposed to LPS but an enhancing trend to those exposed to HS induction effect.

In healthy PBMCs, only LPS pretreated with Gln suppressed mHSP72 baseline expression (Figure 4(a)). Gln when given 1 hour after HS attenuated the HS-induced mHSP72 expression compared to Gln given before induction (Figure 4(b)). In SIRS Gln before LPS further suppressed the already LPS-suppressed mHSP72 baseline expression (Figure 4(c)). Gln before HS showed a trend for stronger effect compared to LPS alone or Gln after LPS (Figure 4(d)). In septic PBMCs, Gln given before LPS abolished the LPS induction effect (Figure 4(e)) on baseline lHSP72 expression. In SS, Gln given before HS enhanced the lHSP72 HS induction (Figure 4(f)).

### 3.5. Related Samples Wilcoxon Signed-Rank Tests of HSP72 mRNA between Gln Treatment Regimens and HS or LPS Induction Methods in H, SIRS, and SS Groups

**Figure 5:**
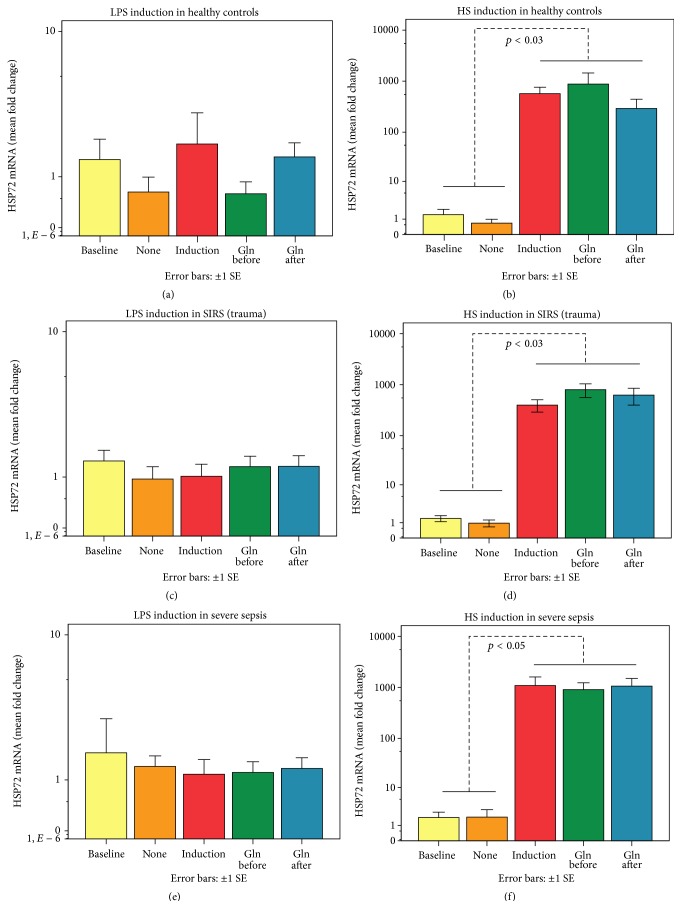
Gln given before or after LPS incubation or HS induction did not modify mRNA in H, SIRS, or SS group (a–f).

When Gln was given before or after LPS incubation (Figures 5(a), 5(c), and 5(e)) or HS induction (Figures 5(b), 5(d), and 5(f)) it could not modify mRNA in H, SIRS, or SS group.

### 3.6. Glutamine Modulating HS and LPS Effects in Individual Patients with Severe Sepsis

**Figure 6:**
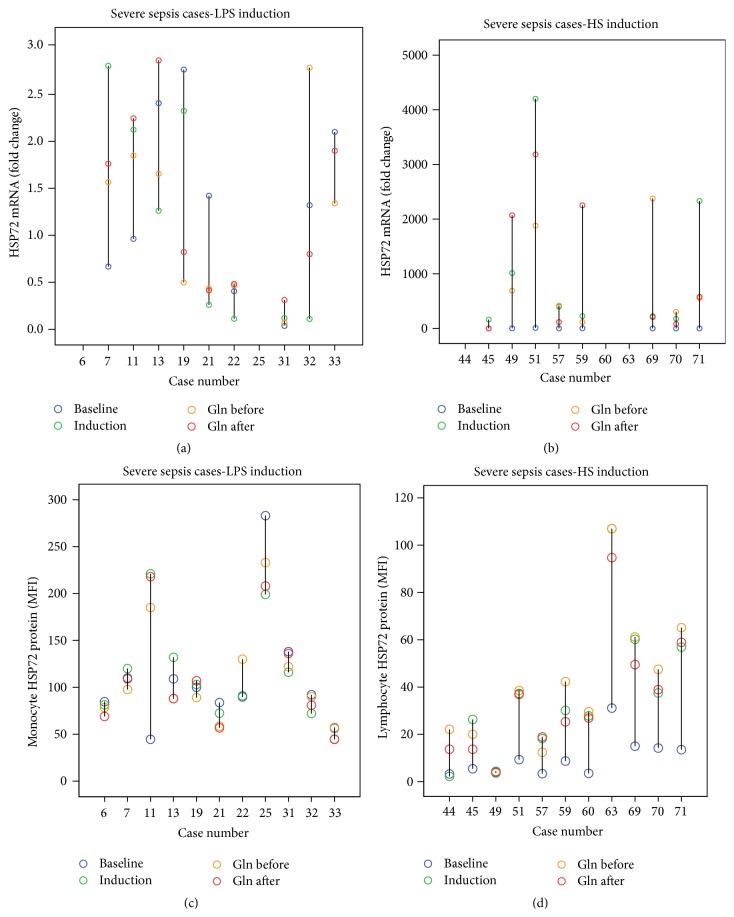
In individual patients with severe sepsis, glutamine effects on either LPS modulating or HS-induced HSP72 mRNA fold change (a-b) or lymphocyte or monocyte HSP72 MFI expression (c-d) were unpredictable.

In individual patients with severe sepsis, glutamine effects on either LPS modulating or HS-induced HSP72 mRNA fold changes (Figures 6(a)-6(b)) and lHSP72 or mHSP72 expressions (Figures 6(c)-6(d)) were unpredictable.

### 3.7. Genetic/Clinical/Serum Cytokines Influence on PBMCs' Baseline mRNA and Protein HSP72 Expression

**Figure 7:**
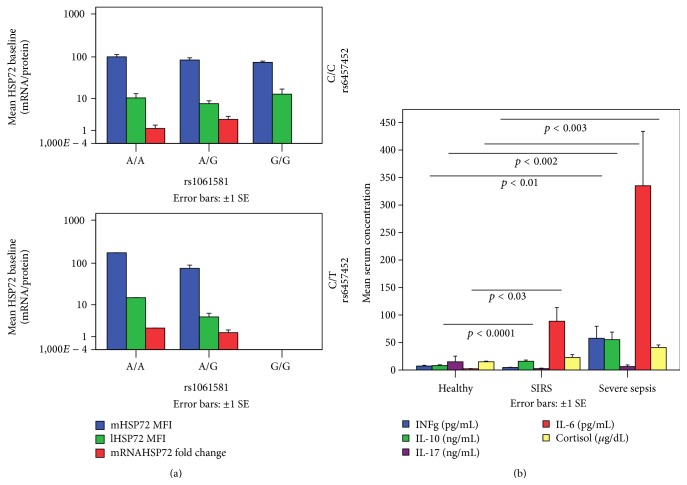
(a) PBMCs' HSP72 mRNA and intracellular lymphocyte and monocyte HSP72 proteins did not differ among haplotypes of the polymorphic rs6457452 and rs1061581 HSP72 SNPs. (b) SS patients had increased serum levels of IL-6, IL-10, INF-*γ*, and cortisol compared to H; SIRS patients had increased serum levels of IL-6 and IL-10 compared to H; differences between SS and SIRS did not reach statistical significance.

The C/C and C/T haplotypes of the polymorphic rs6457452 HSP72 SNP did not differ among groups or between survivors and nonsurvivors. A/A, A/G, and G/G haplotypes of the rs1061581 HSP72 polymorphic SNP also did not differ among groups but G/G haplotype was commonest among nonsurvivors in our sample of patients (*p* < 0.005). Intracellular HSP72 proteins and mRNA did not differ among genotypes (Figure 7(a)).

Groups did not differ regarding age or sex. SS and SIRS patients did not differ regarding the outcome, the severity of illness (APACHE II and SOFA scores), and temperature. SIRS patients had increased serum levels of IL-6 and IL-10 compared to H. SS patients had increased serum levels of IL-6, IL-10, INF-*γ*, and cortisol compared to H (Figure 7(b)). Despite the increased serum levels of cytokines and cortisol in SS compared to SIRS, this difference did not reach statistical significance.

Baseline mRNA was related to serum IL-17 (*r*_*s*_ = 0.84, *p* < 0.0001) and IL-10 (*r*_*s*_ = 0.82, *p* < 0.0001); mHSP72 to serum IL-10 (*r*_*s*_ = 0.37, *p* < 0.004) and INF-*γ* (*r*_*s*_ = 0.46, *p* < 0.002); lHSP72 to serum IL-6 (*r*_*s*_ = 0.37, *p* < 0.02), IL-10 (*r*_*s*_ = 0.53, *p* < 0.0001), INF-*γ* (*r*_*s*_ = 0.90, *p* < 0.0001), and maximum temperature (*r*_*s*_ = 0.40, *p* < 0.02). Neither baseline mRNA nor intracellular HSP72 proteins were related to cortisol serum levels, severity of illness, or patient's age.

## 4. Discussion

We present here the first study to show that intracellular monocyte or lymphocyte HSP72 protein expression and HSP72 mRNA are higher in ICU patients with septic shock/severe sepsis or with trauma compared to healthy individuals. Only neutrophil HSP72 has been previously shown to be increased in septic patients along with inhibited apoptosis [31]. This is also the first report to show that HS but not LPS induces HSP72 mRNA, mHSP72, and lHSP72 of critically ill patients, still preserving a similar PBMCs' response to stress to the one of healthy individuals. In vivo studies have previously shown that 10 days of heat acclimation in healthy individuals increases baseline HSP72, possibly illustrating a PBMC adaptation to HS [32]. It has been also shown that HS-induced HSP72 is blunted after heat acclimation, possibly representing a physiological strain reduction [33].

The LPS repression effect on lHSP72 and mHSP72 has not been reported before in trauma patients, although an LPS dose- and time-dependent inhibition of total HSP72 has been shown in disrupted PBMC of SS patients [34]. In this study, LPS strongly induced IL and/or chemokine PBMC response, revealing different pathways for secreted cytokines and intracellular HSP72 in responding to LPS stress. This opposing response is in accordance with our previous study showing that extracellular supernatant HSP72 was also repressed after LPS induction, contrasting a prompt response of supernatant IL-6 and IL-10 at different time points [23]. IL-8 and MCP-1 were also induced by LPS, contrasting the inert mRNA/l/mHSP72 response and the previously shown IFN-*γ* or IL-17 nonresponse [23]. Similarly, lHSP72 expression determined by immunoblotting in war trauma-exposed patients with posttraumatic stress disorder did not differ from healthy controls [35]. In experimental traumatic brain injury, however, HSP72-depleted mice increased their brain lesion size compared to wild type, suggesting a minimum HSP72-mediated protective effect, leading to decreased intracranial hemorrhage and brain function preservation [36].

Contrary to the LPS suppression effect in SIRS, LPS induction of PBMC's mHSP72 and lHSP72 in severe sepsis may indicate different stress response dynamics of septic and trauma immune cells. We have now shown that HSP72 mRNA exhibits an increasing trend, escalated from H to SIRS and then to SS patients. It seems that the stress response of the central regulator of heat shock gene expression aims to regulate autophagy by inducing the intracellular HSP72 [37]. Thus, the induction of intracellular HSP72 has been considered to be important in acute stress, since it protects cells from imminent danger [38]. This might explain our finding that mHSP72 and lHSP72 expression is higher in ICU patients, compared to healthy individuals, by 4 hours.

A stress response readiness may be indicated in this study by the abrupt induction of both HSP72 mRNA and intracellular HSP72 in healthy and critically ill individuals' PBMCs exposed to near-fatal HS. Hyperthermia (42-43°C) induces endoplasmic reticulum stress and causes protein denaturation/aggregation, resulting in cell apoptosis [39]. In response to fever or heat, however, all living cells respond by synthesizing HSPs, which help cells to protect themselves under stressful conditions [40]. An anti-inflammatory response of human PBMC to HS has been previously demonstrated by inhibiting the LPS-released IL-10 at 43°C [41]. Expanding this finding, we were able to demonstrate an impressive opposing result of HS, promptly inducing HSP72 mRNA and HSP72 proteins but inhibiting IL-6, IL-10, TNF, IL-8, and MCP-1 release in PBMCs' supernatants. In severely ill patients with sepsis, however, the various combined effects of fever or hypothermia and endotoxemia might exert unpredictable dynamically averaged exacerbated attenuated effects on HSP72 mRNA and PBMCs' ILs, making any trial of targeted intervention impossible.

To examine this issue we attempted to relate in vitro baseline HSP72 with in vivo patients' data. The finding that intracellular HSP72 proteins or HSP72 mRNA was related to serum cytokines may indicate a stronger influence of the inflammatory reaction over confounding clinical or hormonal stress responses. Thus, HSP72 mRNA or protein responses were not influenced by cortisol concentration, the severity of illness, or polymorphic HSP72 SNPs. Clinical association results have previously suggested that polymorphic HSP72 SNPs may be responsible for the lower production of intracellular HSP72 inhibiting cytokine inflammatory cellular functions involved in progression to severe sepsis [25]. Results of our small sample size study could not indicate such an influence in any group or treatment modality.

As a mitochondrial substrate, Gln increases the levels of glutathione and malondialdehyde and inhibits reactive oxygen species and inflammation, enhancing cell ATP content [42]. In addition, Gln plays significant role in metabolism of amino acids, replenishes intermediates of the Krebs cycle, improves glucose utilization [43, 44], has anti-inflammatory and immunomodulatory actions, and stimulates HSP72 [45]. Accordingly, Gln supplementation of incubation media increased HSP72 and TNF-*α* release in an in vitro model using blood samples from healthy children [46]. In Gln-depleted murine sepsis, L-Ala-Gln supplementation reestablished intracellular redox status and attenuated the endotoxemia-induced proinflammatory response [47]. Also, the Gln-induced antiapoptotic activity and macrophage modulation prevented tissue damage in malnourished septic rats [48].

A conditionally essential nutrient, Gln induces* Hsf1* gene activation and HSF1 expression by activating its transcription, leading to a robust induction of HSP72 response [49]. Enhancing HSP72 expression, therefore, Gln may protect against a variety of stress cellular insults. Using heparinized whole blood samples from healthy children, Marino et al. have recently shown that Gln supplementation of incubation media promotes HSP72 release [46]. Simply measuring extracellular HSP72, however, it may not be clear whether this release comes from live neutrophils, monocytes, lymphocytes, or dead cells. Working now with specific immune cell populations from healthy individuals and critically ill patients, we demonstrated that when Gln was given before LPS induction it exerted a depressive rather than inductive effect on lymphocyte and monocyte HSP72 proteins of PBMCs. However, under the same experimental conditions, Gln exhibited a trend to enhance further the HS induction effect. Thereby, although Gln pretreatment increases repression after LPS, it enhances induction after HS. Gln after treatment, however, may not modify HSP72 protein induction from monocytes or lymphocytes.

These findings expand results of our previous study showing that 10 mM of L-Gln or L-Ala-Gln without any other stimulation suppresses HSP72 by 4–24 hours [23]. In that study, Gln did not induce any of the Th1, Th2, or Th17 cytokine in either septic or healthy human PBMC. A clinical trial in trauma ICU patients showed that Gln supplementation did not improve the TLR-2 or TLR-4 functional expression in circulating PBMCs or the phagocytic capability [50]. Further expanding these findings, results of this study clearly demonstrate that Gln, under the specific experimental conditions, does not affect HSP72 mRNA in PBMCs of patients with SIRS or SS or healthy individuals as early as 2 hours after exposure to LPS or HS. By its very nature, there are limitations inherent in our experimental study. However reasonable, we tried to touch on the clinical significance derived from results of the present study to the fullest extent possible.

During critical illness, different plasma Gln levels have been reported [51], associated with organ failure [52] or mortality [53]. In addition, it has been recently argued that disrupting the balance of glutamine may have adverse sequences on outcome in patients with critical illness [54]. Thus, randomized studies in infants and children could not replicate experimental results of proposed Gln effects on morbidity and mortality [55]. Reflecting these and other conflicting results, this series' Gln effect on either LPS modulating or HS-induced lHSP72, mHSP72, or mRNA expression in individual SS patients was unpredictable. It has been previously shown that Gln directly induces LPS-stimulated macrophages' TNF-*α* and macrophages' HSP72 expression, while, at the same time, it inhibits peritoneal macrophages' TNF-*α* in murine sepsis [56]. Curiously, in vitro studies have demonstrated that HSP72 mainly exists in the exosomes of B cells and, unexpectedly, on the PBMC's exosomes [57]. All these complex and opposing effects may explain the results of a recent meta-analysis showing that enteral Gln supplementation could not confer clinical benefit in critically ill patients [58].

Overcoming the dose- and time-dependent action, Layered Double Hydroxides- (LDHs-) based systems of drug delivery have recently been demonstrated to deliver better molecules in vitro and in vivo bioactive to cells [59]. Various biomolecules, including amino acids, have been shown to intercalate into or attach on an LDH material surface by reacting through anion exchange or coprecipitation. Thus, diamond nanoparticles with Gln biocomplexes differentiated and enhanced proliferation of chicken embryo pectoral muscle cells [60]. Importantly, critical gate-keepers such as N-glycosylated nutrient transporters may induce overexpression of Golgi enzymes N-acetylglucosaminyl-transferases, increasing the import rate of extracellular Gln into the cell [61]. Thus, nano-Gln and/or Gln enhancers might open up new research pathways, leading to high entry Gln trials in critical illness.

## 5. Conclusions

With clinical and related laboratory data being considered, the results of the present study suggest that intracellular HSP72 proteins and mRNA are activated during severe sepsis or SIRS. They also suggest that heat shock can still enhance monocyte and lymphocyte HSP72 expression, thereby improving host defense in response to fever. Whether this represents a balanced mechanism initiated by the host to counter-balance excessive thermal injury is yet to be elucidated. The failure of LPS to further induce HSP72 in human PBMCs while it promptly triggers a massive cytokine PBMCs' response may suggest alternative pathways bypassing HSP72 innate immune response. An intracellular negative feedback mechanism of the PBMCs' HSP72 cytokine machinery might be also assumed, contrasting an independent positive relation to serum ILs, not being influenced by HSP72 polymorphisms, cortisol levels, or illness severity. Importantly, our data further suggest that glutamine may repress the weak LPS and enhance the strong heat shock induction of monocyte and lymphocyte HSP72 proteins but may not modulate HSP72 mRNA in patients with sepsis or trauma. This glutamine LPS-related repression or HS-related enhancing effect on intracellular HSP72 proteins in ICU patients is reported for the first time. Clearly, future studies, possibly with Gln cell entry enhancers, are needed to better understand the pathways involved in LPS, HS, and Gln modulating effects on HSP mRNA and mHsp72 and lHSP72 protein expression in critical illness.

*What This Study Adds.* This study adds the following:

- Glutamine may repress the monocyte lymphocyte HSP72 proteins exposed to LPS stimulation.
- Glutamine may enhance the strong heat shock induction of monocyte and lymphocyte HSP72 proteins.
- Glutamine may not modulate HSP72 mRNA in patients with sepsis or trauma and healthy individuals.
- Intracellular monocyte or lymphocyte HSP72 expression is higher in critically ill patients with severe sepsis or with trauma compared to healthy individuals.
- Intracellular monocyte or lymphocyte HSP72 expression is induced by heat shock but not by LPS in patients with sepsis or trauma and healthy individuals.
- The cytokines IL-6, IL-8, IL-10, and MCP-1 in PBMCs supernatants are induced by LPS not by heat shock, especially in critically ill patients.
- Induced HSP72 mRNA is related to intracellular HSP72 proteins and is negatively related to supernatant cytokines.
- Intracellular HSP72 proteins and HSP72 mRNA are related to serum cytokines, not significantly being influenced by cortisol response, the severity of illness, and polymorphic HSP72 SNPs.

## Abbreviations

HSP: Heat shock protein
ILs: Interleukins
MCP-1: Monocyte chemoattractant protein-1
Gln: Glutamine
PBMC: Peripheral blood mononuclear cells
SS: Severe sepsis
SIRS: Systemic inflammatory response syndrome
LPS: Lipopolysaccharide
HS: Heat shock
ICU: Intensive care unit.

## References

[1] O'Grady N. P., Barie P. S., Bartlett J. G. (2008). Guidelines for evaluation of new fever in critically ill adult patients: 2008 update from the American College of Critical Care Medicine and the Infectious Diseases Society of America. *Critical Care Medicine*.

[2] Lee B. H., Inui D., Suh G. Y. (2012). Association of body temperature and antipyretic treatments with mortality of critically ill patients with and without sepsis: multi-centered prospective observational study. *Critical Care*.

[3] Young P. J., Saxena M., Beasley R. (2012). Early peak temperature and mortality in critically ill patients with or without infection. *Intensive Care Medicine*.

[4] Li J., Jiang J.-Y. (2012). Chinese Head Trauma Data Bank: effect of hyperthermia on the outcome of acute head trauma patients. *Journal of Neurotrauma*.

[5] Eyers S., Weatherall M., Shirtcliffe P., Perrin K., Beasley R. (2010). The effect on mortality of antipyretics in the treatment of influenza infection: systematic review and meta-analysis. *Journal of the Royal Society of Medicine*.

[6] Niven D. J., Stelfox H. T., Laupland K. B. (2013). Antipyretic therapy in febrile critically ill adults: a systematic review and meta-analysis. *Journal of Critical Care*.

[7] Villar J., Ribeiro S. P., Mullen J. B. M., Kuliszewski M., Post M., Slutsky A. S. (1994). Induction of the heat shock response reduces mortality rate and organ damage in a sepsis-induced acute lung injury model. *Critical Care Medicine*.

[8] Henderson B., Kaiser F. (2013). Do reciprocal interactions between cell stress proteins and cytokines create a new intra-/extra-cellular signalling nexus?. *Cell Stress and Chaperones*.

[9] King Y.-T., Lin C.-S., Lin J.-H., Lee W.-C. (2002). Whole-body hyperthermia-induced thermotolerance is associated with the induction of heat shock protein 70 in mice. *Journal of Experimental Biology*.

[10] Pritchard M. T., Li Z., Repasky E. A. (2005). Nitric oxide production is regulated by fever-range thermal stimulation of murine macrophages. *Journal of Leukocyte Biology*.

[11] Shi Y., Tu Z., Tang D. (2006). The inhibition of LPS-induced production of inflammatory cytokines by HSP70 involves inactivation of the NF-kappaB pathway but not the MAPK pathways. *Shock*.

[12] Kregel K. C. (2002). Invited review: heat shock proteins: modifying factors in physiological stress responses and acquired thermotolerance. *Journal of Applied Physiology*.

[13] Song M., Pinsky M. R., Kellum J. A. (2008). Heat shock factor 1 inhibits nuclear factor-*κ*B nuclear binding activity during endotoxin tolerance and heat shock. *Journal of Critical Care*.

[14] Yoo C.-G., Lee S., Lee C.-T., Kim Y. W., Han S. K., Shim Y.-S. (2000). Anti-inflammatory effect of heat shock protein induction is related to stabilization of I*κ*B*α* through preventing I*κ*B kinase activation in respiratory epithelial cells. *The Journal of Immunology*.

[15] Wischmeyer P. E., Kahana M., Wolfson R., Ren H., Musch M. M., Chang E. B. (2001). Glutamine induces heat shock protein and protects against endotoxin shock in the rat. *Journal of Applied Physiology*.

[16] Wischmeyer P. E., Riehm J., Singleton K. D. (2003). Glutamine attenuates tumor necrosis factor-alpha release and enhances heat shock protein 72 in human peripheral blood mononuclear cells. *Nutrition*.

[17] Ziegler T. R., Ogden L. G., Singleton K. D. (2005). Parenteral glutamine increases serum heat shock protein 70 in critically ill patients. *Intensive Care Medicine*.

[18] Carcillo J. A., Dean J. M., Holubkov R. (2012). The randomized comparative pediatric critical illness stress-induced immune suppression (CRISIS) prevention trial. *Pediatric Critical Care Medicine*.

[19] Heyland D., Muscedere J., Wischmeyer P. E. (2013). A randomized trial of glutamine and antioxidants in critically ill patients. *The New England Journal of Medicine*.

[20] Briassoulis G., Filippou O., Kanariou M., Hatzis T. (2005). Comparative effects of early randomized immune or non-immune-enhancing enteral nutrition on cytokine production in children with septic shock. *Intensive Care Medicine*.

[21] van Zanten A. R. H., Sztark F., Kaisers U. X. (2014). High-protein enteral nutrition enriched with immune-modulating nutrients vs standard high-protein enteral nutrition and nosocomial infections in the ICU: a randomized clinical trial. *The Journal of the American Medical Association*.

[22] Vince R. V., Oliver K., Midgley A. W., McNaughton L. R., Madden L. A. (2010). In vitro heat shock of human monocytes results in a proportional increase of inducible Hsp70 expression according to the basal content. *Amino Acids*.

[23] Briassouli E., Goukos D., Daikos G. (2014). Glutamine suppresses Hsp72 not Hsp90*α* and is not inducing Th1, Th2, or Th17 cytokine responses in human septic PBMCs. *Nutrition*.

[24] Vardas K., Apostolou K., Briassouli E. (2014). Early response roles for prolactin cortisol and circulating and cellular levels of heat shock proteins 72 and 90*α* in severe sepsis and SIRS. *BioMed Research International*.

[25] Temple S. E. L., Cheong K. Y., Ardlie K. G., Sayer D., Waterer G. W. (2004). The septic shock associated HSPA1B1267 polymorphism influences production of HSPA1A and HSPA1B. *Intensive Care Medicine*.

[26] Dellinger R. P., Levy M. M., Rhodes A. (2013). Surviving sepsis campaign: international guidelines for management of severe sepsis and septic shock: 2012. *Critical Care Medicine*.

[27] Basi D. L., Ross K. F., Hodges J. S., Herzberg M. C. (2003). The modulation of tissue factor by endothelial cells during heat shock. *The Journal of Biological Chemistry*.

[28] Coëffier M., Marion R., Ducrotté P., Déchelotte P. (2003). Modulating effect of glutamine on IL-1 *β*-induced cytokine production by human gut. *Clinical Nutrition*.

[29] Singleton K. D., Beckey V. E., Wischmeyer P. E. (2005). Glutamine prevents activation of NF-*κ*B and stress kinase pathways, attenuates inflammatory cytokine release, and prevents acute respiratory distress syndrome (ARDS) following sepsis. *Shock*.

[30] Vargas-Alarcón J. D., Londoño G., Hernández-Pacheco R. (2002). Heat shock protein 70 gene polymorphisms in Mexican patients with spondyloarthropathies. *Annals of the Rheumatic Diseases*.

[31] Hashiguchi N., Ogura H., Tanaka H. (2001). Enhanced expression of heat shock proteins in activated polymorphonuclear leukocytes in patients with sepsis. *Journal of Trauma—Injury, Infection and Critical Care*.

[32] Yamada P. M., Amorim F. T., Moseley P., Robergs R., Schneider S. M. (2007). Effect of heat acclimation on heat shock protein 72 and interleukin-10 in humans. *Journal of Applied Physiology*.

[33] McClung J. P., Hasday J. D., He J.-R. (2008). Exercise-heat acclimation in humans alters baseline levels and ex vivo heat inducibility of HSP72 and HSP90 in peripheral blood mononuclear cells. *The American Journal of Physiology—Regulatory Integrative and Comparative Physiology*.

[34] Schroeder S., Bischoff J., Lehmann L. E. (1999). Endotoxin inhibits heat shock protein 70 (HSP70) expression in peripheral blood mononuclear cells of patients with severe sepsis. *Intensive Care Medicine*.

[35] Matić G., Milutinović D. V., Nestorov J. (2014). Mineralocorticoid receptor and heat shock protein expression levels in peripheral lymphocytes from war trauma-exposed men with and without PTSD. *Psychiatry Research*.

[36] Kim J. Y., Kim N., Zheng Z., Lee J. E., Yenari M. A. (2013). The 70 kDa heat shock protein protects against experimental traumatic brain injury. *Neurobiology of Disease*.

[37] Dokladny K., Zuhl M. N., Mandell M. (2013). Regulatory coordination between two major intracellular homeostatic systems: heat shock response and autophagy. *The Journal of Biological Chemistry*.

[38] Jacquier-Sarlin M. R., Fuller K., Dinh-Xuan A. T., Richard M.-J., Polla B. S. (1994). Protective effects of hsp70 in inflammation. *Experientia*.

[39] Bettaieb A., Averill-Bates D. A. (2015). Thermotolerance induced at a mild temperature of 40°C alleviates heat shock-induced ER stress and apoptosis in HeLa cells. *Biochimica et Biophysica Acta*.

[40] Lin L.-C., Chen H.-W., Yang R.-C. (2005). Expression of Hsp72 in lymphocytes in patients with febrile convulsion. *Kaohsiung Journal of Medical Sciences*.

[41] Bouchama A., Hammami M. M., Al Shail E., DeVol E. (2000). Differential effects of in vitro and in vivo hyperthermia on the production of interleukin-10. *Intensive Care Medicine*.

[42] Ziegler T. R., Benfell K., Smith R. J. (1990). Safety and metabolic effects of L-glutamine administration in humans.. *Journal of Parenteral and Enteral Nutrition*.

[43] Brunengraber H., Roe C. R. (2006). Anaplerotic molecules: current and future. *Journal of Inherited Metabolic Disease*.

[44] Owen O. E., Kalhan S. C., Hanson R. W. (2002). The key role of anaplerosis and cataplerosis for citric acid cycle function. *Journal of Biological Chemistry*.

[45] Roth E. (2008). Nonnutritive effects of glutamine. *Journal of Nutrition*.

[46] Marino L. V., Pathan N., Meyer R., Wright V. J., Habibi P. (2014). The effect of 2 mMol glutamine supplementation on HSP70 and TNF-*α* release by LPS stimulated blood from healthy children. *Clinical Nutrition*.

[47] Cruzat V. F., Bittencourt A., Scomazzon S. P., Leite J. S. M., De Bittencourt P. I. H., Tirapegui J. (2014). Oral free and dipeptide forms of glutamine supplementation attenuate oxidative stress and inflammation induced by endotoxemia. *Nutrition*.

[48] Oliveira G. P., Silva J. D., de Araújo C. C. (2014). Intravenous glutamine administration reduces lung and distal organ injury in malnourished rats with sepsis. *Shock*.

[49] Xue H., Slavov D., Wischmeyer P. E. (2012). Glutamine-mediated dual regulation of heat shock transcription factor-1 activation and expression. *The Journal of Biological Chemistry*.

[50] Pérez-Bárcena J., Crespí C., Regueiro V. (2010). Lack of effect of glutamine administration to boost the innate immune system response in trauma patients in the intensive care unit. *Critical Care*.

[51] Heyland D. K., Dhaliwal R. (2013). Role of glutamine supplementation in critical illness given the results of the REDOXS Study. *Journal of Parenteral and Enteral Nutrition*.

[52] Rodas P. C., Rooyackers O., Hebert C., Norberg Å., Wernerman J. (2012). Glutamine and glutathione at ICU admission in relation to outcome. *Clinical Science*.

[53] Straaten H. M. O.-V., Bosman R. J., Treskes M., van der Spoel H. J. I., Zandstra D. F. (2001). Plasma glutamine depletion and patient outcome in acute ICU admissions. *Intensive Care Medicine*.

[54] Bistrian B. R. (2013). Glutamine and antioxidants in critically ill patients. *The New England Journal of Medicine*.

[55] Briassouli E., Briassoulis G. (2012). Glutamine randomized studies in early life: the unsolved riddle of experimental and clinical studies. *Clinical and Developmental Immunology*.

[56] Liang M., Wang X., Yuan Y., Zhou Q., Tong C., Jiang W. (2009). Different effect of glutamine on macrophage tumor necrosis factor-alpha release and heat shock protein 72 expression in vitro and in vivo. *Acta Biochimica et Biophysica Sinica*.

[57] Johnson J. D., Fleshner M. (2006). Releasing signals, secretory pathways, and immune function of endogenous extracellular heat shock protein 72. *Journal of Leukocyte Biology*.

[58] van Zanten A. R. H., Dhaliwal R., Garrel D., Heyland D. K. (2015). Enteral glutamine supplementation in critically ill patients: a systematic review and meta-analysis. *Critical Care*.

[59] Zhang K., Xu Z. P., Lu J. (2014). Potential for layered double hydroxides-based, innovative drug delivery systems. *International Journal of Molecular Sciences*.

[60] Grodzik M., Sawosz F., Sawosz E. (2013). Nano-nutrition of chicken embryos. The effect of in ovo administration of diamond nanoparticles and L-glutamine on molecular responses in chicken embryo pectoral muscles. *International Journal of Molecular Sciences*.

[61] Rahman A. M. A., Ryczko M., Nakano M. (2015). Golgi *N*-glycan branching *N*-acetylglucosaminyltransferases I, V and VI promote nutrient uptake and metabolism. *Glycobiology*.

